# Unproved Remedies

**Published:** 1881-08

**Authors:** P. P. Wells

**Affiliations:** Brooklyn, N. Y.


### HOMŒOPATHIC PHYSICIAN.

A MONTHLY JOURNAL OF MEDICAL SCIENCE.

"If our school ever gives up the strict inductive method of Hahnemann, we are lost, and deserve to be mentioned only as a caricature, in the history of medicine."—CONSTANTINE HERING.

Vol. I. AUGUST, 1881. No. 8.

### UNPROVED REMEDIES.

P. P. WELLS, M. D., BROOKLYN, N. Y.

(*Read before Kings County Homœopathic Medical Society.*)

THE difficulty of giving exact definitions of facts has been felt by all who have given much of time or effort to gain precise knowledge of any natural science. To cover the whole ground, leaving out no essential element; to give this in the fewest possible words, avoiding all which gives no aid to a right and clear understanding of the fact in hand; to so select the terms employed that they shall express neither more nor less than is contained in the subject to be defined—has been recognized in all times by all students who have made thoroughness a characteristic of their work. It is but little less difficult to state in brief terms the elements of a science, or a system of philosophy, or to give rules of practice under a system of philosophy, without superfluous words, or omission of some element more or less necessary to make the rule or rules complete. To do this requires that the mind shall be so habitually familiar with the subject in hand that it readily takes in its whole, and its parts, whenever turned to its contemplation. In attempting this it will sometimes happen that in endeavors to bring rules to their briefest expression some one, of more or less importance, is overlooked.

It is a matter for regret that in the declaration of the "International Hahnemannian Association," which was intended to present to us the homœopathic law with its necessary corollaries, an exceedingly important member of those corollaries should have been omitted. It does not state in the last of these corollaries, as it should, that the "minimum dose of the dynamized drug" should be also of a drug which *has been proved* on the healthy organism, before it can be given for the cure of the sick, as required by our law.

This omission may have come from so clear a consciousness of the self-evident necessity of this proving that its mention was deemed uncalled for. If this were the occasion of its absence from the declaration it was clearly a mistake. This appears from the right claimed by some, who profess faith in, and true allegiance to, our law, to give their patients, as curatives, substances which have not been proved on the healthy organism, as our law requires that every substance shall be before it can be known as the simillimum the cure requires. Not only so, but they claim that cures are made by these unproved substances, and that in a manner superior to those effected by medicines which have been proved and given as the simillimum of the case under treatment.

It is not the intent of this paper to question the truth or ability of those who claim that cures are made by these unproved substances, but to protest against their being incorporated into *homœopathic* records. And also against homœopathy being held in any degree responsible for them, as we regard practice with these as wholly foreign to the philosophy and practice of this system as taught by the master; as really so, as is the poly-pharmacy of the allopath.

And more than this is claimed for this departure from the homœopathic law, viz.: that this proceeding is in accordance with *law*, as is the administration of the simillimum under the homœopathic law. What this new law is which justifies this use of unknown agents for the cure of the sick, we are not told, and of its existence we have only recently been informed. When given to us in terms as explicit as those which declare the homœopathic law, we can be in a position to judge better of its value than we are now, when all we know of it is that it is claimed to *be*, and that those who set up this claim declare that they can do better for the sick, under its guidance, with means of which they know little or nothing of their effects upon the healthy organism, than they can under the homœopathic law, with agents which have been fully proved, and therefore are known as to their action on the healthy. This, we believe, is the newest claim set up for the superiority of ignorance over knowledge. If we gain nothing more from this last choice of darkness rather than light, we are likely to receive from it an impression, as to the ability of these advocates of a new law, as specific prescribers under the guidance of the old.

It is notable that this claim for superior success under a law of therapeutics, not yet made known, and from the use of agents unproved, and therefore wholly unavailable in practice under any law, is put forward by professed believers in the law of similars as nature's law of healing; and more than this, that their own practice is homœopathic, even when under the control of this unknown law and when employing these unknown means.

Let us see what ground, if any, they have for this claim for the homœopathicity of their practice while employing unknown agents—if in their use they are complying with the requirements of any law. If so, it cannot be that which demands that all medicines used under its authority shall first be proved on the healthy organism, that its effects may be known, and so *known* to be similar in these effects to the phenomena of the case to be treated. How can it be known of any unproved agent whether its effects are like to those phenomena or not? It should be remembered, while we are considering this claim for the homœopathicity of a practice which employs unknown means, that homœopathy requires that *all* agents employed in accordance with its law of similars, shall first have been given to healthy persons, that by the actions of them so learned, we may know whether that which we employ is like; without such proving it is impossible to know this. In the absence of this, to claim for any drug that it is homœopathic to any given case is demonstrative of sheer ignorance of what homœopathy really is; or, of a bold impudence in claiming the honors of its law for that which, at best, is but rank empiricism.

In the reference we have made to practice with unproved agents, it will be readily perceived that we are alluding chiefly to the use of some morbid products that have been potentized and given to the sick, without the proving on the healthy, which our law requires of all agents before it sanctions their use as curatives. It may be replied, and it has been said, that the disease giving us this potentized product is a proving of the product, and of a kind the most perfect. At first glance this certainly has a seeming of truth on the face of it, and as truth is all we want, we will see how this claim for proving will bear examination.

It will be said that the disease producing this potentized agent was the result of a specific poison. Granted. That the poison is present in the product. Granted. Therefore, the phenomena of the disease are the expression of the action of the poison on the living organism, and, therefore, we have in them the most perfect of all provings. This conclusion is hasty and inadmissible. Something more than making an individual sick by giving him a drug is necessary for a proving of that drug. We require, first of all, that the prover shall be in sound health at the time he takes the drug; and second, that the resulting phenomena be recorded at the time of their occurrence, with all their concomitants and modalities, with utmost exactness and detail, and this to the end of the prover's suffering. All this, and many repetitions of this, are required before any drug can be regarded as *proved* in the manner our law requires. Anything less than this, it will not accept as a proving.

Now take one of these unproved substances, *Syphilinum*, for example. The substance potentized is the product of syphilis: syphilis is the result of a specific poison. Now, who has kept such a record of the daily and hourly experiences of the poisoned person with all the concomitants and modalities, as is required in proving drugs, as will give us their experience in the proper form for a drug-proving of any medicinal substance? Has this been done in the case of the disease from which the potentized morbid product was obtained? If not, then we have no proving of this product, in the unrecorded phenomena developed in the life of the poisoned individual.*

We, as homœopathists, have nothing by which we can determine whether this potentized poison is, in its nature, as revealed in the experience of the patient, at all like the phenomena of any case for which, on the testimony of these advocates of the new law, and unproved remedies, we might be tempted to give it. In the absence of the required record, we may as well understand that both we and these advocates, for the purposes of specific prescribing, know nothing at all about it.

And then, our law requires that the prover of drugs, for our use, shall be in perfect health when he enters on his duties as a prover. This is needful, that there may come to us, in the recorded effects of the drug, no mixture whatever of any old cachexias. Such a

* Even if there be such a record of any given case of the disease, and a portion of its poisonous product has been potentized, this record can only be available as a guide for the clinical use of this nosode as obtained from this particular case. The record of any other case, if made, may so differ from this, because of constitutional or circumstantial peculiarities, as to render its product so unlike that of the first record as to render it wholly unsuitable as a guide for the clinical use of this. The same is true of the product of any other example of the disease.

mixture would render a proving rather a curse than a help to us in our clinical duties. Now, how is this in the case of syphilitic patients? Are they the persons whom we should most readily accept as free from all other diseased taints than that of the specific poison, or are they more likely than others to show cachectic complications? Where these are present, how are they to modify the nature of the morbid product we are asked to accept, as proved by the experience of the patient? Does not the liability to such complications destroy the uniformity of the nature of the preparations made from different specimens of the disease? This uniformity is regarded as of the utmost importance in drugs to be proved. Why less in the case of nosodes?*

In short, the further we pursue the matter of this class of unproved, so-called, remedies, the more and greater difficulties we meet in the way of their intelligent incorporation into a rational, sound, homœopathic practice. Indeed, as at present informed, this seems wholly impracticable. It would seem that the homœopathy of Hahnemann has active opponents and obstacles enough to encounter, to warrant its calling on all its adherents to see to it that it is spared from being handicapped with new heresies, and, above all, from that of practicing with unproved agents.

But, it is said, practice with these unproved agents "*is in accordance with a* LAW, *which law is a fixed fact*," etc. The first suggestion which occurs to us after reading this assertion, is to inquire: in accordance with what law, and who was the maker of the law which assures us greater practical success from the use of the unknown, than can be realized from that which *is* known?

Till these questions are answered satisfactorily, certainly in our present state of knowledge it cannot be otherwise than pardonable, if we regard this alleged new law of therapeutics as having its origin and its existence, very much, if not wholly, in the imagination

* We require of drugs, before we accept them for proving, that they be perfect specimens of their kind, and pure, *i. e.*, unmixed with other drugs or substances, which can, in any way or degree, modify their action on the person, in order that we have the pure effects on the organism of the substance proved. Now, in the case of these nosodes, it would seem impossible that we can have any assurance of this purity. Dyscrasias are so common and so various in the bodies of mankind, and especially, in that class in which the diseases from which these nosodes are obtained they are most frequently met, and the power of these to modify the nature and product of the original disease so wholly unknown, that the product must, of necessity, be always impure, in the sense in which we require purity in drugs to be proved, *i. e.*, the product must always be a mixture, and a mixture ever varied by the various nature of the associate dyscrasia; so that a uniformity of the product to be potentized is an impossibility, and, therefore, its use must be ever uncertain and unsafe.

of those who seem to be its sole advocates. We have, outside of this new discovery, a law of therapeutics which we claim to be of divine enactment, because of its universal adaptability to the pains and sicknesses of our race. We claim for it that it is equal to all our needs as healers. That when it was made a part of the laws of our being its Author knew well what He was about, and being able to do this, He made our law so perfect and comprehensive that no other was needed, and that, so far as we know, in all His works, He has never dealt with nor created superfluities.* And therefore in accordance with this general plan, having given one law which is equal to all our needs, He has never enacted a second. This seems to us reasonable and in perfect harmony with what we know of the other works of the Author of the homœopathic law. There may be other laws, but it is certain we know no other, and we get along right royally well in the absence of any knowledge of them. Though it is admitted that other therapeutic laws may exist, we do not believe they do, because we have no need of them. What we need for a more ready and perfect cure of the sick is not additional laws of healing, but a more thorough comprehension of the one God-given law, and a more perfect acquaintance with the agents which are proved and ready for our use; and a more intelligent and diligent study of these and of the elements of the sicknesses to be cured; and a more careful selection of the curatives this law requires, and then all the needs felt by indolence or ignorance, of other laws or means, will vanish away, and the one law, and the means it requires and employs, will stand justified by practical results before men, even before those who are sometimes wrecked by imaginations more active than intelligent.

---

*The Fifth Annual Session of the Missouri Institute of Homœopathy*, will be held at Sweet Springs, Mo., August 3d and 4th, 1881. Members and their families will be entertained at the Sweet Springs Hotel, at $2 per day. These Springs are growing into great favor, and a large and very interesting meeting is expected.

WM. D. FOSTER, M. D., *Secretary.*

---

* Natural laws require no supplements nor duplicates. Each is complete in itself for the purposes for which it was created. Who is there so insane as to imagine the law of gravitation requiring or admitting of supplements or substitution.

